# Twice recurrent gallstone ileus: a case report

**DOI:** 10.1186/1752-1947-6-362

**Published:** 2012-10-24

**Authors:** Rhys Jones, Daniel Broman, Richard Hawkins, David Corless

**Affiliations:** 1 Department of Surgery, Leighton Hospital, Middlewich Road, Crewe, Cheshire, CW1 4QJ, UK; 2 Department of Radiology, Leighton Hospital, Middlewich Road, Crewe, Cheshire, CW1 4QJ, UK

**Keywords:** Recurrent gallstone ileus, enterotomy, fistulectomy

## Abstract

**Introduction:**

Gallstone ileus is a rare cause of bowel obstruction and results from the passage of gallstones into the bowel.

**Case presentation:**

We present the case of an 83-year-old Caucasian woman who had three episodes of gallstone ileus, each of which was managed with simple enterotomy. This sequence is one of the first reported in the medical literature and may be seen to challenge the traditional surgical approach of enterotomy alone.

**Conclusions:**

The available evidence comparing enterotomy alone with combined enterotomy, cholecystectomy, and fistula closure in the management of gallstone ileus is reviewed. Neither approach is clearly identified as superior, but available series suggest that simple enterotomy may be safer than a combined approach and does not result in a higher rate of recurrent biliary disease.

## Introduction

Gallstone ileus occurs when gallstones migrate from the gallbladder to the bowel through a cholecysto-enteric fistula, causing a mechanical obstruction. Investigation of suspected cases with abdominal computed tomography (CT) will generally show gallstones in the bowel lumen with proximal dilatation. Patients are often frail and elderly, and so surgical treatment is generally limited to stone retrieval through an incision in the bowel wall known as an enterotomy [1]. Owing to the risk of recurrent gallstones and associated complications, some surgeons advocate a “one-stage” repair comprising cholecystectomy and fistula closure in addition [2]. We present the case of a patient who had three episodes of gallstone ileus and a review of the evidence comparing these two operative techniques.

## Case presentation

An 83-year-old Caucasian woman with rheumatoid arthritis and a large hiatus hernia was admitted with a one-day history of severe abdominal pain and vomiting. She was tachycardic, tachypnoeic, and pyrexial. Abdominal examination revealed tenderness and peritonism to her right iliac fossa. Her bowel sounds were diminished. Serology revealed a white blood cell count of 16.1g/dL, and plain abdominal radiographs revealed no obvious perforation or obstruction. A differential diagnosis of appendicitis and cecal carcinoma was considered, and the decision to operate was made. At laparotomy through a lower midline incision, palpation of the small bowel revealed that a large impacted gallstone in the mid-ileum was causing patchy mesenteric necrosis. There was free fluid in the right iliac fossa but no perforation. A 12cm length of ileum was resected, and a primary stapled anastomosis was performed. The gallbladder was found to be packed with stones. No fistula was identified, but the dissection was limited by dense fibrosis.

**Figure 1:**
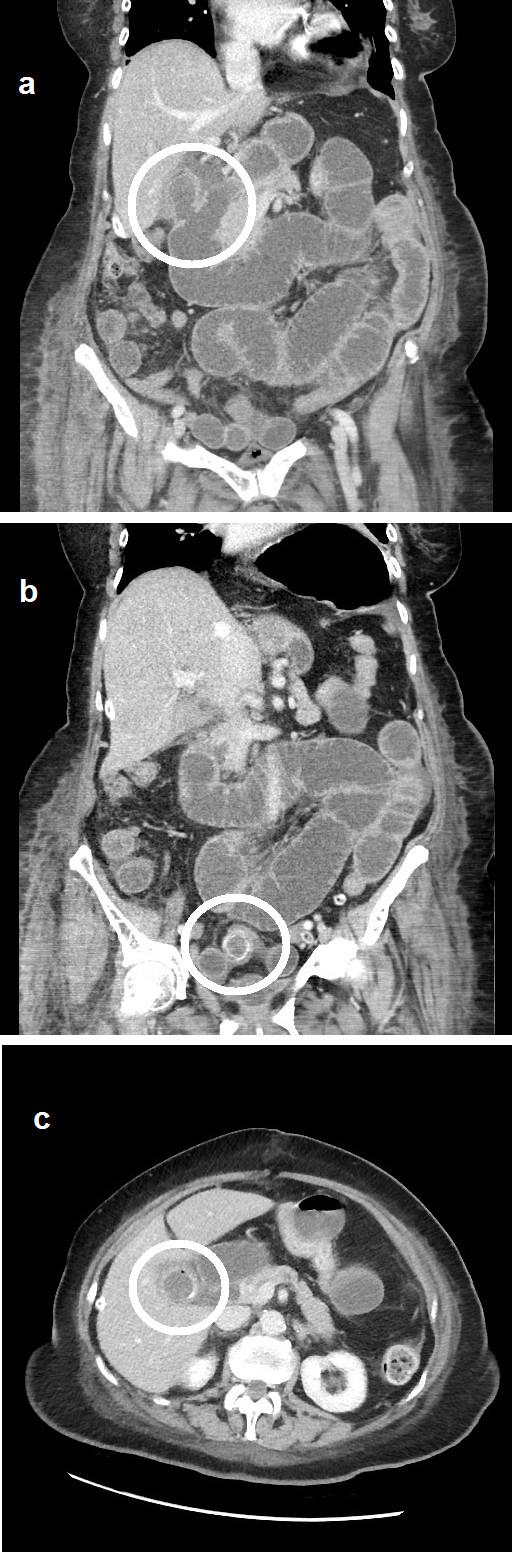
**Computed tomography scan performed after the initial laparotomy.** (**a**) Coronal computed tomography image of a cholecysto-duodenal fistula. (**b**) Coronal computed tomography image of small bowel calculus with proximal dilatation. (**c**) Axial computed tomography image of an additional calculus in the gallbladder.

After initial signs of recovery, our patient developed worsening abdominal pain on the fourth post-operative day, and abdominal CT was undertaken (Figure 1). The scan revealed a low-grade small bowel obstruction due to a laminated ileal gallstone, a fistula between the gallbladder and duodenum, and a further stone in the gallbladder. After a trial of conservative management, a second laparotomy on day nine revealed a gallstone impaction in the distal small bowel and a contained leak in the original ileal anastomosis. The stone was removed through a transverse enterotomy, and the anastomosis was refashioned with sutures. The inflammatory mass in the right upper quadrant, with the residual gallbladder stone, was palpable but not disturbed. The post-operative recovery was complicated by a *Clostridium difficile* infection, but our patient returned home to independent living four weeks after the second procedure.

**Figure 2:**
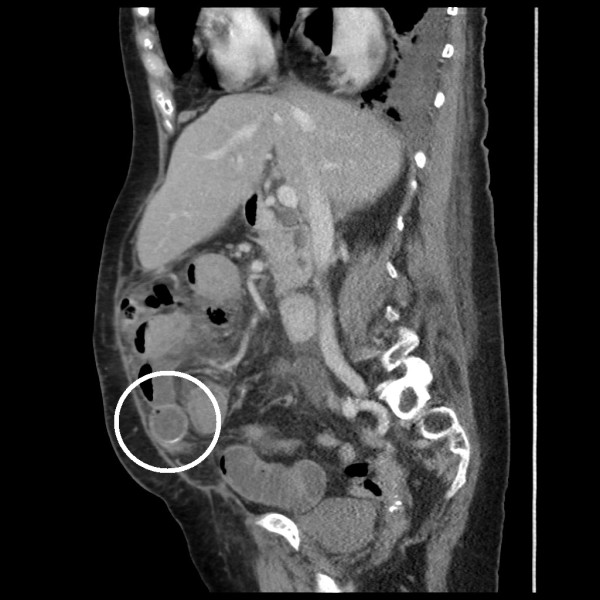
Sagittal computed tomography image shows a calculus in the distal small bowel with proximal dilatation.

Twelve months later, she presented with a further episode of central abdominal pain associated with vomiting and distension. An ultrasound scan revealed a contracted gallbladder with a 10mm common bile duct, and intravenous antibiotics were commenced for a presumed cholecystitis. She failed to settle, and a CT scan revealed free intraperitoneal gas and fluid, a persistent cholecysto-duodenal fistula, and a further gallstone obstructing the small bowel. This gallstone corresponded in size and shape to that seen in the gallbladder 12 months earlier (Figure 2). At laparotomy, a limited small bowel resection was performed for perforation proximal to a gallstone obstruction. The stone was removed, and a hand-sewn anastomosis was performed. Her post-operative recovery was again complicated by a *C. difficile* infection in addition to a prolonged period of respiratory failure managed with a tracheostomy in critical care. She was discharged to her own home six weeks after surgery and made a good recovery. Two months after discharge, magnetic resonance cholangiopancreatography did not visualize any residual gallbladder stones.

## Discussion

Gallstone ileus accounts for around 25% of mechanical small bowel obstruction in patients over 65 years of age [3]. A history of biliary symptoms is variable and the obstruction can be intermittent, and so diagnosis is often delayed. Generally patients are women and often are frail and face a post-operative mortality rate of more than 10% [4].

Operative management is challenging. Simple enterotomy risks further stones and the potential complications of cholangitis, pancreatitis, and recurrent gallstone ileus. Conversely, cholecystectomy and fistula repair within dense adhesions risk duodenal injury and biliary leak. Both options have an associated mortality in the population in question.

In the case described, it is unclear whether the stone removed at the second laparotomy was in the gallbladder or the bowel lumen at the time of the first operation. Twelve months later, the CT images suggest that a further stone had migrated into the bowel. Our experience demonstrates the importance of inspecting the entirety of the small bowel at the time of the initial laparotomy and illustrates that late recurrence of gallstone ileus, albeit rare, can occur [5].

To the best of our knowledge, this is only the third reported case of twice recurrent gallstone ileus [6,7]. The recent timing of the two previous reports suggests that the phenomenon is becoming more common, presumably as a result of the increasing age of the population, and prompted us to revisit the evidence comparing enterotomy alone with the one-stage approach in the initial management of the condition.

To date, the largest review of reported cases of gallstone ileus, which consisted of 1001 cases, was published by Reisner and Cohen in 1994 [1]. The findings are frequently quoted, but the work is limited by the heterogeneity of the reports analyzed. We undertook an electronic literature search to identify all subsequent published comparisons of enterotomy alone versus one-stage repair in the management of gallstone ileus. The terms “gallstone ileus” and “gallstone obstruction” were inserted into PubMed, reference lists were checked, and Scopus (ScienceDirect) was used to identify the citations of key articles. We identified eight comparative studies, of which three comprised at least five patients in each arm. The smaller studies were excluded. Three primary endpoints of operative mortality, operative morbidity, and recurrent biliary disease were considered. Operative complications were defined as those preceding discharge from hospital. Recurrent biliary disease is defined as cholecystitis, choledocholithiasis, cholangitis, recurrent gallstone ileus, pancreatitis, or cholangiocarcinoma during follow-up. The findings of the three studies identified, in addition to those of Reisner and Cohen, are presented in Table 1. 

**Table 1:** Comparative studies in the management of gallstone ileus

			**Enterotomy**				**One-stage repair**				**Comparison**		
**Authors**	**Year**	**Follow-up (range) in months**	**N**	**Operative mortality**	**Operative morbidity**	**Recurrent biliary disease**	**N**	**Operative mortality**	**Operative morbidity**	**Recurrent biliary disease**	**Operative mortality**	**Operative morbidity**	**Recurrent biliary disease**
Reisner and Cohen [1]	1994	NR	801	11.7%	NR	NR (6% RGSI)	113	16.9%	NR	NR (5.3% RGSI)	Favors E (NS)	NR	NR
Rodriguez-Sanjuan *et al*. [4]	1997	40 (4–96)	16	18.8%	NR	6.3%	9	33.3%	NR	22.2%	Favors E (NS)	NR	Favors E (NS)
Doko *et al*. [8]	2003	0.5 (0–2)	11	9.1%	27.3%	NR	18	11.1%	61.1%	NR	Favors E (NS)	Favors E (P = 0.043)	NR
Tan *et al*. [9]	2004	24 (8–56)	7	0.0%	57.1%	0.0%	12	0.0%	58.3%	0.0%	Equal	Favors E (NS)	Equal

*E* enterotomy, *N* number, *NR* not reported, *NS* non-significant, *RGSI* recurrent gallstone ileus.

In their combined series, Reisner and Cohen [1] published operative mortality rates of 16.9% for one-stage repair and 11.7% for enterolithotomy (P <0.17). The three more recent series also suggest a non-significant excess in operative mortality following one-stage repair. Where reported, comparison of morbidity between the two procedures appears to favor enterotomy and this finding was statistically significant in the study by Doko *et al*. [8]. There are no data comparing one- or two-stage repair with simple enterotomy in the context of recurrent gallstone ileus. Interestingly, none of these papers reported an increased frequency of recurrent biliary disease at follow-up. They do, however, illustrate two important points: the high levels of comorbidity in this patient group and the increased operating times associated with one-stage repair [8,9].

These studies all describe small, retrospective, non-randomized series without predetermined follow-up. Given the rarity of the presentation, the inconsistency of pre-operative diagnosis, and the frailty of many patients, formal randomized analysis is unlikely to provide a more definitive set of answers in the future.

## Conclusions

Gallstone ileus is a surgical emergency affecting a high-risk population. Although there remains no strong evidence in favor of enterotomy alone or one-stage repair, the available data suggest that enterotomy is safe and not associated with increased long-term biliary complications.

## Consent

Written informed consent was obtained from the patient for publication of this case report and accompanying images. A copy of the written consent is available for review by the Editor-in-Chief of this journal.

## Abbreviation

CT: Computed tomography.

## Competing interests

The authors declare that they have no competing interests.

## Authors’ contributions

DB collated patient data relevant to the case report. RJ reviewed the medical history, wrote the manuscript, and performed the literature review. RH reviewed the radiology and contributed to the writing of the manuscript. DC contributed to the writing of the manuscript. All authors read and approved the final manuscript.
